# Effects of IMB model-based rehabilitation on exercise compliance and knee function after total knee arthroplasty: a randomized controlled trial

**DOI:** 10.3389/fmed.2025.1619435

**Published:** 2025-12-15

**Authors:** Zhenqi Wei, Yanhong Wei, Yanping Feng, Qu Yang, Li Zhang, Xiaohong He

**Affiliations:** 1 Department of Joint Surgery, The People’s Hospital of SND, Suzhou, Jiangsu, China; 2 Department of ENT, The People’s Hospital of SND, Suzhou, Jiangsu, China; 3 Department of Nursing, The people’s Hospital of SND, Suzhou, Jiangsu, China; 4 Department of Orthopedics, The People’s Hospital of SND, Suzhou, Jiangsu, China

**Keywords:** arthroplasty, rehabilitation, patient compliance, information-motivation-behavioral skills model, knee joint

## Abstract

**Objective:**

To evaluate the effectiveness of rehabilitation training measures under the Information-Motivation-Behavioral Skills (IMB) model on rehabilitation training compliance and knee function of patients after total knee arthroplasty (TKA).

**Methods:**

A randomized controlled trial was performed. A total of 90 patients who underwent TKA at our hospital between June 2022 and June 2023 were randomly assigned to either a control group (*n* = 45, conventional nursing care), or an observation group (*n* = 45, rehabilitation training based on the IMB model). Patient compliance (Orthopedic Functional Exercise Compliance Scale), knee joint function (Hospital for Special Surgery (HSS) Knee Rating Scale), symptoms and functional status [the Knee Injury and Osteoarthritis Outcome Score (KOOS)], pain intensity [Visual Analog Scale (VAS)], and exercise tolerance [the 6-min walk distance (6MWD) test] were assessed at discharge, as well as at 1 and 3 months postoperatively.

**Results:**

A significant Group × Time interaction *(p* < 0.001) for all outcomes, indicating different recovery trajectories between groups. While groups were comparable at discharge (*p* > 0.05), the IMB-based intervention group demonstrated significantly greater improvement than the control group at both 1 and 3 months. Specifically, the observation group had higher functional exercise compliance (1-month: 60.89 vs. 50.11; 3-month: 70.07 vs. 61.87), better knee function (HSS: 1-month: 72.31 vs. 62.04; 3-month: 80.82 vs. 69.29; KOOS also significantly higher), lower pain scores (VAS: 1-month: 3.89 vs. 4.82; 3-month: 2.00 vs. 3.84), and longer 6MWD (1-month: 416.02 m vs. 373.09 m; 3-month: 530.82 m vs. 459.67 m) (all between-group *p* < 0.001).

**Conclusion:**

Rehabilitation training interventions based on the IMB model can significantly enhance patients’ compliance with postoperative rehabilitation exercises and effectively improve knee joint function, reduce pain intensity, and enhance exercise tolerance, demonstrating the practical utility and value of the IMB model in promoting functional recovery after TKA.

## 1 Introduction

Total knee arthroplasty (TKA) is an effective treatment for severe knee disease, particularly in patients with severe osteoarthritis (1). This surgery relieves pain, improves joint function, and improves the quality of life by removing damaged joint structures and implanting prostheses (2). Long-term follow-up data show that the excellent rate of TKA is as high as 85–95%, and the prosthesis survival rate can reach 90–95% for more than 15 years (3). However, despite these favorable surgical results, a major barrier to achieving optimal functional recovery is poor patient compliance with postoperative rehabilitation exercises. Many patients experience discomfort, fear, or lack of motivation, leading to suboptimal implementation of rehabilitation protocols. Such non-adherence can compromise the overall effectiveness of the surgery and delay recovery. Therefore, identifying and applying effective rehabilitation models is crucial to improving adherence and promoting better postoperative outcomes (4, 5).

Traditional postoperative rehabilitation approaches often focus on providing patients with instructions and general guidance. However, these methods may fail to address patients’ psychological barriers, lack of intrinsic motivation, or insufficient self-management skills, leading to poor adherence to rehabilitation exercises. In response to these limitations, the Information-Motivation-Behavioral Skills (IMB) model has gained attention in recent years for its effectiveness in promoting health-related behaviors (6). The underlying assumption of the IMB model is that change in health behavior requires three key factors: information, motivation, and behavioral skills (7, 8). Information refers to specific knowledge related to health behaviors, including the hazards of disease, preventive measures, etc.; motivation includes motivations at the individual and societal levels, such as attitudes toward healthy behaviors and self-efficacy; behavioral skills refer to the skills and abilities that individuals need to perform healthy behaviors. The IMB model has demonstrated its effectiveness in various rehabilitation contexts. For example, the IMB model has been widely used in cardiac rehabilitation care, and has significantly improved the rehabilitation outcomes of patients with coronary heart disease through motivational interviewing and behavioral skills guidance (9). In addition, in rehabilitation nursing after hip arthroplasty, the IMB model-based intervention program significantly improved patients’ compliance with rehabilitation training, promoted joint function recovery, and relieved pain, hinting its potential effects on rehabilitation of TKA (10). However, the application of the IMB model in TKA rehabilitation remains underexplored. This gap in the literature highlights the need for further investigation into how the IMB model can be effectively utilized to improve postoperative rehabilitation outcomes for TKA patients.

Therefore, in this article, the IMB model rehabilitation exercise measures were taken for patients after total knee arthroplasty in the hospital, and the effects of improving the compliance of rehabilitation exercises and knee joint function were studied via a randomized controlled trail.

## 2 Materials and methods

### 2.1 General data

**FIGURE 1:**
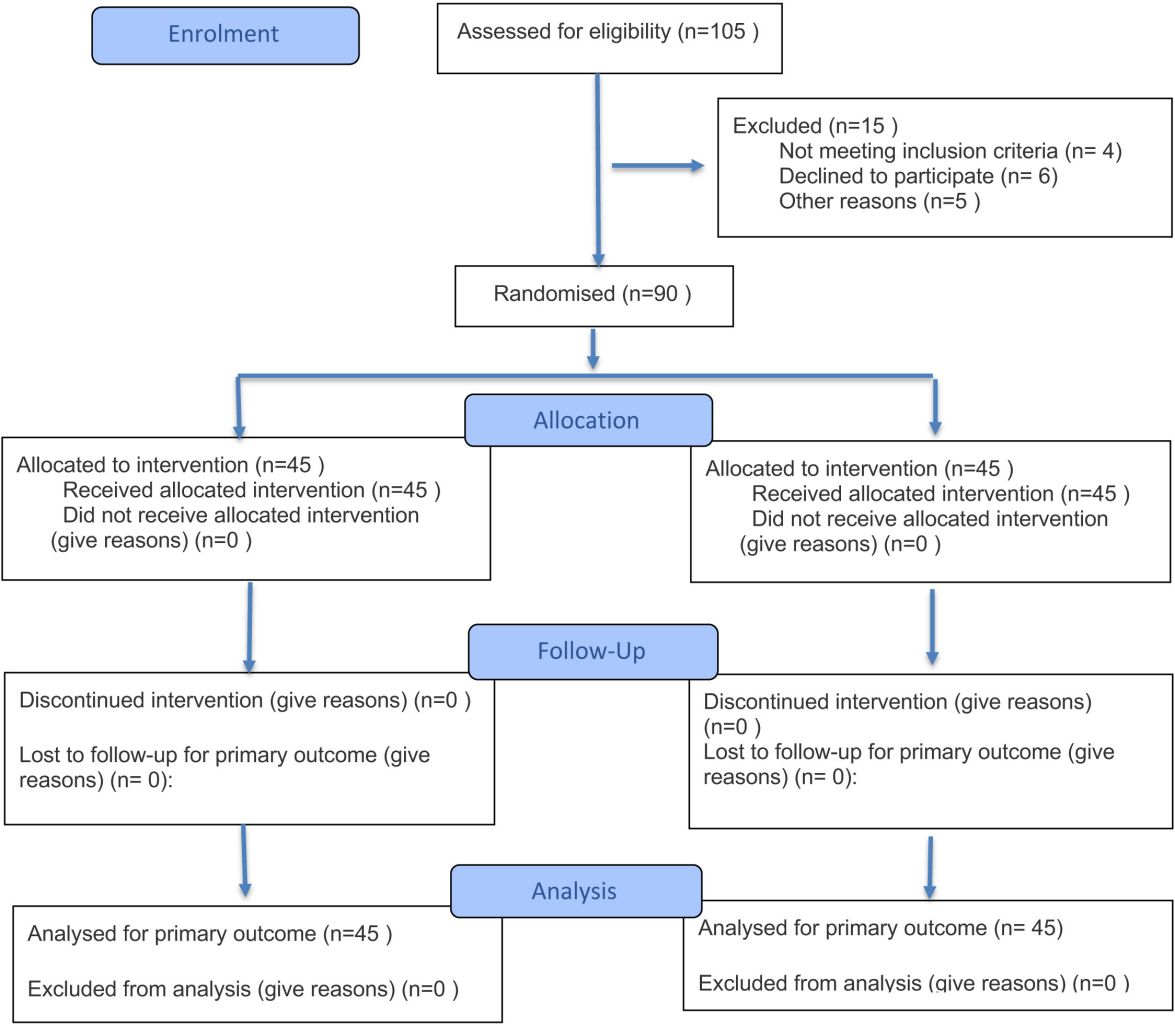
Flow diagram of our study.

A total of 90 patients treated with total knee arthroplasty in the hospital from June 2022 to June 2023 were randomly divided into a control group (*n* = 45, intervention with conventional nursing measures) and an observation group (*n* = 45, rehabilitation training intervention based on the IMB model) (Figure 1). Inclusion criteria: (1) Patients who met the diagnostic criteria for patients with knee osteoarthritis according to the American Academy of Orthopaedic Surgeons (11); (2) Patients who had the surgical indications of unilateral total knee arthroplasty; (3) Patients who were able to understand, express and read correctly; (4) Patients and their families gave informed consent to the study and signed the informed consent form. Exclusion criteria: (1) Patients who withdrew during the experiment; (2) Patients with mental disorders or cognitive impairment; (3) Patients with severe systemic diseases; (4) Patients with severe organ diseases.

### 2.2 Methods

This study was conducted in accordance with the declaration of Helsinki. This study was conducted with approval from the Ethics Committee of The People’s Hospital of SND (No. 2025-133). Written informed consent was obtained from all participants. This study was registered at https://www.isrctn.com (No. ISRCTN12515586).

The sample size was calculated using a two-sided test with a significance level (α) of 0.05 and a power (1−β) of 0.80, based on expected differences in the primary outcome between the two groups. The estimated minimum required sample size was 41 patients per group. To account for an anticipated dropout or loss to follow-up rate of 10%, the final sample size was adjusted to 45 patients per group, resulting in a total of 90 participants.

Then, participants were randomly assigned to either the observation group or the control group in a 1:1 ratio using a computer-generated random number sequence. The randomization sequence was generated by an independent statistician using SPSS version 26.0 (IBM Corp., Armonk, NY, United States) and was concealed in sequentially numbered, opaque, sealed envelopes. Upon enrollment, each participant was assigned the next envelope in order, ensuring allocation concealment. Although blinding of participants and intervention providers was not feasible due to the nature of the intervention, outcome assessors and data analysts were blinded to group assignments to minimize assessment and analytical bias.

The patients in the control group were intervened with routine nursing measures after being admitted to the hospital. Health education was carried out by the primary nurse in accordance with the requirements of rehabilitation training and intervention after knee arthroplasty, and the patient’s family was informed to actively participate in the learning of relevant knowledge. After the surgery, the patient’s vital signs need to be strictly tested, and after the patient regains consciousness and maintains a stable condition, the patient is instructed on how to correctly use the walker to carry out training, and the patient is guided to get out of bed as soon as possible. After the surgery, it is also necessary to pay attention to functional exercise interventions such as continuous passive exercise, straight leg raise exercise, ankle pump exercise, and home rehabilitation guidance and discharge health education intervention should be carried out when the patient is discharged from the hospital. Specific exercise methods: Postoperative days 0–3: Ankle pumps (10–15 cycles per session, 3 sessions daily); Quadriceps isometric contractions (10 reps/set with 5-s holds, 3 sets daily); Straight leg raises (5–8 reps/set, 2 sets daily); Continuous passive motion (CPM) initiated on postoperative day 2 (0–30°, 30 min once daily). Days 4–7: Bedside sitting (5 min/session, 3 sessions daily); Walker-assisted ambulation (5–10 m/session, twice daily); Active knee flexion (target 60°, 3 sets daily). Discharge criteria (days 7–10): Active knee flexion ≥ 60°, independent walker use, no signs of wound infection. Post-Discharge: Standard rehabilitation manual distribution, monthly outpatient follow-ups, recommended 30-min daily training.

The observation group of patients was intervened with IMB model rehabilitation training measures on the basis of conventional nursing measures. (1) Intervention team: IBM model rehabilitation training management team was composed of 1 primary nurse, 1 psychologist, 1 rehabilitation therapist, 1 orthopedic surgeon, and 1 orthopedic specialist nurse. A specialist nurse was responsible for the implementation, and the specific implementation of the rehabilitation guidance program was summarized and analyzed through weekly meetings. (2) Information support: ➀ Information transfer and education: Develop a detailed rehabilitation exercise plan and provide clear guidance, including the type, frequency, intensity, and duration of exercise. Provide the patient with detailed information about rehabilitation exercises, explaining the positive effects of exercise on rehabilitation and the negative consequences that may result from not doing it. ➁ Personalized information support: Understand the patient’s background, health status, and rehabilitation goals to provide personalized information support. Provide relevant information and advice on the specific barriers that patients may face. ➂ Use multimedia resources: make educational videos, charts, or manuals to show the correct way to perform rehabilitation exercises in an intuitive and vivid way. Use cellphone apps or online platforms to provide regularly updated information to keep patients interested in rehabilitation. ➃ Regular communication: Establish regular communication channels, such as telephone, email, or online chat, to answer questions that patients may have and provide additional information support. Through regular follow-up and feedback, the rehabilitation plan is adjusted in a timely manner to ensure that patients have a clear understanding of their progress. ➄ Establish a community support system: Encourage patients to participate in rehabilitation communities or groups to share experiences, receive support, and increase the exchange of information. Provide social media platforms or online forums where patients can encourage each other and share their recovery journeys. (3) Motivational interview: ➀ Building a relationship of trust: At the beginning of the interview, make sure to establish a good relationship of trust with the patient so that the patient feels respected and supported. Understand the patient’s recovery goals, expectations, and concerns to personalize the interview. ➁ Open-ended questions and listening: Ask open-ended questions and encourage patients to share their views and feelings about rehabilitation exercises. By listening to the patient’s responses, their motivation and willingness to recover are understood. ➂ Emphasizing personal values: Guide patients to review their lives and values to emphasize the importance of rehabilitation exercises to achieve their important goals. To explore the value of rehabilitation in improving quality of life and maintaining independence. Addressing motivational disorders: Understand the motivational disorders that the patient may face, such as fears, doubts, or slackness, and work together to find solutions. Discuss past successful rehabilitation experiences to inspire confidence and motivation in the patient. Using open-ended questioning method to verify patient motivation in Motivational Interviewing. (4) Behavioral skills training: ➀ Design a personalized rehabilitation exercise plan: Work with the patient to develop a personalized rehabilitation exercise plan that meets the rehabilitation needs and goals. Make sure the rehabilitation plan is specific, including the type, intensity, frequency, and duration of the exercise. ➁ Provide specific and clear guidance: Provide detailed guidance to ensure that patients understand the correct way to perform each rehabilitation exercise action. Use illustrations, demonstrations, or videos to help patients understand and imitate. ➂ Phased goal setting: Set short-term and long-term rehabilitation exercise goals with patients to ensure that the goals are specific, quantifiable, and achievable. Break down the big goals into small ones to help patients gradually achieve their long-term recovery goals. According to the exercise plan below, verify whether the patient has achieved it and make timely adjustments.

Specific exercise methods: Early postoperative rehabilitation (days 0–3) requires patients to perform ankle pumps (10–15 repetitions/hour of dorsiflexion-plantarflexion cycles) for venous return enhancement, concurrently with quadriceps isometric contractions in supine position: 5–10-s holds with full knee extension, repeated 10–15 times per set for 3–5 daily sets, while straight leg raises demand elevating the affected limb to 30°–45° while maintaining knee extension, holding for 5 s before controlled lowering at 10 repetitions/set across 3 daily sets; additionally, continuous passive motion (CPM) initiation on postoperative day 1 starts at 0°–30° range, administered twice daily for 30-min sessions with progressive flexion increases exceeding 90°.(2) Intermediate rehabilitation (4–14 days after surgery): Beginning on the 3rd–4th day after surgery, patients are instructed to use a walker to stand beside the bed and walk short distances (5–10 m), gradually increasing to twice daily, with each session lasting 10-15 minutes. Starting from the 5th day after surgery, patients are guided to transition from a lying position to a seated position beside the bed, with both legs naturally hanging down, to promote knee flexion. (3) Home rehabilitation after discharge (2–6 weeks post-surgery): With the assistance of handrails or crutches, start by stepping up with the healthy leg, then the affected leg, gradually increasing the height and frequency of steps. Starting from 4 weeks post-surgery, perform balance training, single-leg standing (holding onto the back of a chair), and center of gravity transfer training, with 2 sets per day and 10 repetitions per set. (The differences of Rehabilitation Programs between the Two Groups are shown in Supplementary Table S1).

To ensure the fidelity of the IMB model intervention, the following measures were implemented:

(1) Staff training and assessment:

All intervention team members received standardized training, followed by competency assessments through simulated interactions and written examinations (passing threshold: ≥ 85%).

(2) Structured adherence checklist:

A predefined checklist was utilized to document the completion of core intervention steps (including information delivery, motivational interviewing, and skill training) during each session, with regular audits by team leaders.

(3) Random audio evaluations:

Approximately 20% of motivational interviewing and skill-training sessions were randomly selected for independent expert evaluation using the Motivational Interviewing Treatment Integrity (MITI) 4.2 scale.

(4) Patient feedback:

At 1-month post-intervention, patients provided feedback on the intervention’s clarity, supportiveness, and skill guidance via a 5-point Likert scale questionnaire.

### 2.3 Observation indicators

All the indicators were measured by a single orthopedic surgeon who did not participate in this study. (1) Functional exercise compliance: The functional exercise compliance of the two groups was evaluated by the Orthopedic Functional Exercise Compliance Scale (12) at the time of discharge, 1 month after surgery, and 3 months after surgery, respectively, mainly to investigate compliance with physical exercise, compliance with active learning and exercise, and psychological compliance with exercise; a scoring method of 1∼5 scores was selected, with the highest score of 75, and the score value and the patient’s compliance with functional exercise are in a proportional relationship. The scale has been validated with good reliability and validity (Cronbach’s α = 0.930, content validity index = 0.936).

(2) Knee joint function score: At the time of discharge, 1 month after surgery, and 3 months after surgery, the knee function evaluation of the two groups was completed through the Knee HSS Rating Scale (13), mainly to evaluate knee joint stability, flexion deformity, muscle strength, range of motion (ROM), function, pain and other items, with a maximum score of 100, and the score was directly proportional to the patient’s knee joint function. In addition, Knee Injury and Osteoarthritis Outcome Score (KOOS) was also used to evaluate the symptoms and functions, including pain, other symptoms, function in daily living, function in sport and recreation, and knee-related quality of life.

(3) Pain intensity: Assessed using the Visual Analog Scale (VAS) with a 10-cm scale ranging from “no pain” to “worst pain,” where patients mark their perceived pain level.

(4) Exercise Tolerance: The 6-min walk distance (6MWD) test was used to assess functional capacity. Participants were instructed to walk as far as possible on a flat, hard surface within 6 min, with self-paced speed adjustments and rest permitted as needed. The total distance covered (in meters) was recorded. Heart rate and oxygen saturation (SpO_2_) were monitored throughout the test, which was immediately terminated if participants experienced chest pain, dyspnea, or other discomfort.

### 2.4 Statistical analysis

Statistical analyses were performed using SPSS Statistics (version 24.0; IBM Corp., Armonk, NY, United States) and R (version 4.3.0; R Foundation for Statistical Computing, Vienna, Austria). All analyses were conducted based on the intention-to-treat (ITT) principle. Descriptive statistics are presented as mean ± standard deviation (SD) for continuous variables and as frequency (n,%) for categorical variables. The normality of the distribution for continuous variables was assessed using the Shapiro-Wilk test. Between-group comparisons of baseline characteristics were performed using independent samples *t*-tests for normally distributed continuous variables, Mann-Whitney U tests for non-normally distributed variables, and Chi-square (χ^2^) tests (or Fisher’s exact test where appropriate) for categorical variables. For the primary analysis of longitudinal outcomes (functional exercise compliance, HSS, KOOS, ROM, VAS, and 6MWD), which involved repeated measures at discharge, 1 and 3 months, a linear mixed model (LMM) was employed—as appropriately suggested by the reviewers—to account for within-subject correlations over time. The models included fixed effects for Group (Observation vs. Control), Time (as a categorical factor), and the Group × Time interaction, and a random intercept for subject ID. Model parameters were estimated using restricted maximum likelihood (REML). The assumptions of normality and homoscedasticity of the model residuals were visually inspected and confirmed. If the Group × Time interaction was statistically significant, *post hoc* pairwise comparisons were conducted at each time point with Bonferroni adjustment for multiple comparisons. Effect sizes are reported as Cohen’s d for pairwise comparisons (calculated from the LMM-estimated mean differences and pooled standard deviation) and partial eta-squared (η^2^) for the F-tests of fixed effects from the LMM, providing a measure of the variance explained by each factor. Results from the LMM are presented as estimated marginal means ± standard error (SE), along with mean differences and their 95% confidence intervals (CIs) where applicable. A two-sided *p*-value < 0.05 was considered statistically significant for all analyses.

## 3 Results

### 3.1 General data

The control group consisted of 45 patients (25 males and 20 females), aged 30–60 years, with a mean age of 47.62 ± 3.25 years. The observation group also included 45 patients (24 males and 21 females), aged 30–60 years, with a mean age of 48.44 ± 3.92 years. The mean disease duration was 5.44 ± 0.50 years in the control group and 5.58 ± 0.50 years in the observation group, with a disease course ranging from 3 to 10 years in both groups. The Control group comprised 14 subjects with junior high school or lower education, 16 with high school/vocational training, and 15 with college or higher degrees, while observation group included 13, 18, and 14 subjects in these respective categories. Surgical sites included 23 left knees and 22 right knees in the control group, and 22 left knees and 23 right knees in the observation group.

There were no statistically significant differences between the two groups in terms of gender, age, disease duration, educational level, annual income, residence, or surgical site (*P* > 0.05), indicating baseline comparability (Table 1).

**TABLE 1:** Comparison of baseline data of the two groups of patients [$\bar{x}$ ± S/n(%)].

Index	Observation group *(n* = 45)	Control group *(n* = 45)	t/*x*^2^	*P*
Gender/male	24(53.33)	25(55.56)	0.045	0.832
Age (95% CI)	48.44 ± 3.92	47.62 ± 3.25	1.083	0.282
Surgical site			0.044	0.833
Left knee	22(48.89)	23(51.11)	1.262	0.210
Right knee	23(51.11)	22(48.89)		
Course of disease	5.58 ± 0.50	5.44 ± 0.50		
Education level			0.189	0.910
Junior high school or lower education	13(28.89)	14(31.11)	2.133	0.545
High school/vocational training	18(40.00)	16(35.56)		
College or higher degrees	14(31.11)	15(33.33)		
Annual income (10k CNY)				
< 1	10 (22.22%)	12 (26.67%)	0.178	0.673
1–5	18 (40.00%)	17 (37.78%)		
5–10	11 (24.44%)	9 (20.00%)		
> 10	6 (13.33%)	7 (15.56%)		
Residence				
Urban	25 (55.56%)	23 (51.11%)		
Rural	20 (44.44%)	22 (48.89%)		

### 3.2 Functional exercise compliance score before and after intervention

The results of the linear mixed model analysis for functional exercise compliance are presented in Tables 2, 3. There was a statistically significant main effect of Time [*F*(2, 176) = 1250.48, *p* < 0.001, partial η^2^ = 0.93] and Group [*F*(1, 88) = 150.25, *p* < 0.001, partial η^2^ = 0.63]. Crucially, a significant Group × Time interaction was observed [*F*(2, 176) = 85.16, *p* < 0.001, partial η^2^ = 0.49], indicating that the trajectory of improvement in compliance scores over the study period differed significantly between the two intervention groups. *Post hoc* analyses with Bonferroni adjustment revealed that while the two groups were comparable at discharge (mean difference = 0.22, 95% CI: –1.09 to 1.53, *p* > 0.99), the observation group demonstrated significantly higher compliance scores than the control group at both the 1-month (mean difference = 10.78, 95% CI: 9.13–12.43, *p* < 0.001) and 3-month (mean difference = 8.20, 95% CI: 6.55–9.85, *p* < 0.001) follow-ups. Furthermore, within each group, scores increased significantly between each consecutive assessment time point (all *p* < 0.001).

**TABLE 2:** Functional exercise compliance scores: estimated marginal means and group comparisons.

Group	At discharge (T0)	1 month (T1)	3 months (T2)
Control (*n* = 45)	40.60 ± 0.46	50.11 ± 0.60	61.87 ± 0.60
Observation (*n* = 45)	40.82 ± 0.53	60.89 ± 0.63	70.07 ± 0.60
Mean difference (95% CI) (Observation-Control)	0.22 (–1.09 to 1.53)	10.78 (9.13–12.43)	8.20 (6.55–9.85)

Data are presented as Estimated Marginal Means ± Standard Error (SE) derived from a linear mixed model. The mean difference (and its 95% Confidence Interval, CI) represents the advantage of the Observation group over the Control group at each time point, based on model-based pairwise comparisons with Bonferroni adjustment for multiple comparisons. The analysis was performed on the intention-to-treat (ITT) population. Normality of residuals was confirmed by visual inspection of Q-Q plots. CI, confidence interval.

**TABLE 3:** Linear mixed model (fixed effects) for functional exercise compliance scores.

Effect	*F*-value	df1, df2	*P*-value	Effect size (partial η^2^)
Group	150.25	1, 88	< 0.001	0.63
Time	1250.48	2, 176	< 0.001	0.93
Group × Time	85.16	2, 176	< 0.001	0.49

The Group effect indicates a significant overall difference between the Observation and Control groups across all time points. The Time effect indicates that scores changed significantly across the measurement points. The significant Group × Time interaction effect indicates that the pattern of change in compliance scores over time was significantly different between the two groups (see Table 2 for *post hoc* comparisons). df, degrees of freedom.

### 3.3 Knee HSS score before and after intervention

The results for knee joint function, as measured by the HSS, KOOS, and range of motion (ROM), are presented in Tables 4–9. Linear mixed model analysis revealed a significant Group × Time interaction effect for the HSS score [*F*(2, 176) = 182.37, *p* < 0.001; Table 5], KOOS score [*F*(2, 176) = 67.45, *p* < 0.001; Table 7], and knee ROM [*F*(2, 176) = 9.87, *p* < 0.001; Table 9], indicating that the improvement trajectories for all three outcomes differed significantly between the two groups.

**TABLE 4:** Knee HSS scores: estimated marginal means and group comparisons.

Group	At discharge (T0)	1 month (T1)	3 months (T2)
Control (*n* = 45)	59.09 ± 0.41	62.04 ± 0.35	69.29 ± 0.57
Observation *(n* = 45)	58.49 ± 0.39	72.31 ± 0.54	80.82 ± 0.71
Mean difference (95% CI) (Observation-Control)	–0.60 (–1.63 to 0.43)	10.27 (9.08–11.46)	11.53 (10.17–12.89)

Data are presented as Estimated Marginal Means ± Standard Error (SE) derived from a linear mixed model. The mean difference (and its 95% CI) represents the advantage of the Observation group over the Control group at each time point, based on model-based pairwise comparisons with Bonferroni adjustment. The analysis was performed on the intention-to-treat (ITT) population. Normality of residuals was confirmed by visual inspection of Q-Q plots. HSS, Hospital for Special Surgery Knee Rating Scale; CI, confidence interval.

**TABLE 5:** Linear mixed model results: tests of fixed effects for knee HSS score.

Effect	*F*-value	df1, df2	*P*-value	Effect size (partial η^2^)
Group	315.65	1, 88	< 0.001	0.78
Time	892.14	2, 176	< 0.001	0.91
Group × Time	182.37	2, 176	< 0.001	0.67

The significant Group × Time interaction effect indicates that the pattern of change in HSS scores over time was significantly different between the two groups (see Table 4 for *post hoc* comparisons).

**TABLE 6:** KOOS scores: estimated marginal means and group comparisons.

Group	At discharge (T0)	1 month (T1)	3 months (T2)
Control (*n* = 45)	68.62 ± 0.47	76.51 ± 0.42	89.60 ± 0.43
Observation (*n* = 45)	69.22 ± 0.44	84.11 ± 0.42	95.42 ± 0.25
Mean difference (95% CI) (Observation-Control)	0.60 (–0.62 to 1.82)	7.60 (6.43–8.77)	5.82 (5.01–6.63)

Data are presented as Estimated Marginal Means ± Standard Error (SE) derived from a linear mixed model. The mean difference (and its 95% CI) represents the advantage of the Observation group over the Control group at each time point, based on model-based pairwise comparisons with Bonferroni adjustment. The analysis was performed on the intention-to-treat (ITT) population. Normality of residuals was confirmed by visual inspection of Q-Q plots. KOOS, Knee Injury and Osteoarthritis Outcome Score; CI, confidence interval.

**TABLE 7:** Linear mixed model results: tests of fixed effects for KOOS score.

Effect	*F*-value	df1, df2	*P*-value	Effect size (partial η^2^)
Group	198.74	1, 88	< 0.001	0.69
Time	2450.88	2, 176	< 0.001	0.97
Group × Time	67.45	2, 176	< 0.001	0.43

The significant Group × Time interaction effect indicates that the pattern of change in KOOS scores over time was significantly different between the two groups (see Table 6 for *post hoc* comparisons).

**TABLE 8:** Knee range of motion (ROM): estimated marginal means and group comparisons.

Group	At discharge (T0)	1 month (T1)	3 months (T2)
Control (*n* = 45)	72.84 ± 1.51	91.89 ± 1.08	98.04 ± 0.91
Observation (*n* = 45)	74.11 ± 1.64	97.78 ± 0.83	101.44 ± 0.76
Mean difference (95% CI) (Observation-Control)	1.27 (–2.98 to 5.52)	5.89 (3.47–8.31)	3.40 (1.58–5.22)

Data are presented as Estimated Marginal Means ± Standard Error (SE) derived from a linear mixed model. The mean difference (and its 95% CI) represents the advantage of the Observation group over the Control group at each time point, based on model-based pairwise comparisons with Bonferroni adjustment. ROM is measured in degrees (°). The analysis was performed on the intention-to-treat (ITT) population. Normality of residuals was confirmed by visual inspection of Q-Q plots. ROM, range of motion; CI, confidence interval.

**TABLE 9:** Linear mixed model results: tests of fixed effects for knee ROM.

Effect	*F*-value	df1, df2	*P*-value	Effect size (partial η^2^)
Group	35.18	1, 88	< 0.001	0.29
Time	658.33	2, 176	< 0.001	0.88
Group × Time	9.87	2, 176	< 0.001	0.10

The significant Group × Time interaction effect indicates that the pattern of change in knee ROM over time was significantly different between the two groups (see Table 8 for *post hoc* comparisons).

*Post hoc* analyses confirmed that the two groups were comparable at discharge for all functional measures (all *p* > 0.05). However, at both the 1 and 3-month follow-ups, the Observation group demonstrated statistically significant and clinically relevant improvements compared to the Control group, with higher HSS and KOOS scores and greater knee ROM (all *p* < 0.01; Tables 4, 6, 8).

### 3.4 Comparison of pain scores and 6-min walk distance between groups at different time points

The results for pain intensity (VAS) and functional capacity (6MWD) are presented in Tables 10–13. The linear mixed model analysis revealed a significant Group × Time interaction effect for both VAS [*F*(2, 176) = 45.22, *p* < 0.001; Table 11] and 6MWD [*F*(2, 176) = 92.15, *p* < 0.001; Table 13], indicating that the patterns of pain reduction and improvement in walking capacity over time differed significantly between the two intervention groups.

**TABLE 10:** Pain intensity (VAS): estimated marginal means and group comparisons.

Group	At discharge (T0)	1 month (T1)	3 months (T2)
Control (*n* = 45)	5.73 ± 0.20	4.82 ± 0.17	3.84 ± 0.14
Observation (*n* = 45)	6.02 ± 0.22	3.89 ± 0.17	2.00 ± 0.12
Mean difference (95% CI) (Observation-Control)	0.29 (–0.27 to 0.85)	–0.93 (–1.38 to –0.48)	–1.84 (–2.17 to –1.51)

Data are presented as Estimated Marginal Means ± Standard Error (SE) derived from a linear mixed model. The mean difference (and its 95% CI) represents the difference in VAS scores (Observation minus Control). Lower scores indicate less pain. Analyses are based on model-based pairwise comparisons with Bonferroni adjustment. VAS, Visual Analog Scale; CI, confidence interval.

**TABLE 11:** Linear mixed model results: tests of fixed effects for VAS scores.

Effect	*F*-value	df1, df2	*P*-value	Effect size (partial η^2^)
Group	105.33	1, 88	< 0.001	0.55
Time	285.74	2, 176	< 0.001	0.76
Group × Time	45.22	2, 176	< 0.001	0.34

The significant Group × Time interaction effect indicates that the pattern of pain reduction (change in VAS scores) over time was significantly different between the two groups (see Table 10 for *post hoc* comparisons).

**TABLE 12:** Six-min walk distance (6MWD): estimated marginal means and group comparisons.

Group	At discharge (T0)	1 month (T1)	3 months (T2)
Control (*n* = 45)	303.07 ± 2.76	373.09 ± 2.59	459.67 ± 1.93
Observation (*n* = 45)	306.40 ± 2.51	416.02 ± 1.79	530.82 ± 3.30
Mean difference (95% CI) (Observation-Control)	3.33 (–3.58 to 10.24)	42.93 (38.30– 47.56)	71.15 (66.18–76.12)

Data are presented as Estimated Marginal Means ± Standard Error (SE) derived from a linear mixed model. The mean difference (and its 95% CI) represents the advantage in meters of the Observation group over the Control group at each time point, based on model-based pairwise comparisons with Bonferroni adjustment. The analysis was performed on the intention-to-treat (ITT) population. Normality of residuals was confirmed by visual inspection of Q-Q plots. 6MWD, 6-min walk distance; CI, confidence interval.

**TABLE 13:** Linear mixed model results: tests of fixed effects for 6MWD.

Effect	*F*-value	df1, df2	*P*-value	Effect size (partial η^2^)
Group	402.18	1, 88	< 0.001	0.82
Time	2540.50	2, 176	< 0.001	0.97
Group × Time	92.15	2, 176	< 0.001	0.51

The significant Group × Time interaction effect indicates that the pattern of improvement in functional capacity (change in 6MWD) over time was significantly different between the two groups (see Table 12 for *post hoc* comparisons). The very large effect size for Group (Partial η^2^ = 0.82) indicates a substantial effect of the intervention on walking distance.

*Post hoc* comparisons showed no significant differences between the groups at discharge for either VAS or 6MWD (*p* > 0.05) (Tables 10, 12). However, at the 1- and 3-month follow-ups, the Observation group demonstrated significantly superior outcomes compared to the Control group, with substantially lower pain scores (mean differences at T1 and T2: –0.93 and –1.84, respectively; both *p* < 0.001) and markedly longer distances walked in 6 min (mean differences at T1 and T2: 42.93 and 71.15 m, respectively; both *p* < 0.001).

## 4 Discussion

Early rehabilitation training after total knee arthroplasty (TKA) is indeed limited by a variety of factors, including tolerance, general condition, mental state, and age. Together, these factors influence patients’ adherence to functional exercises, especially in the early postoperative period (14). Studies have shown that severe postoperative pain can lead to a fear of any behavior that may trigger pain and to resist early functional exercise (15). Scientific rehabilitation training can prevent joint stiffness, muscle atrophy, and thrombosis, while improving joint function and quality of life (16). However, due to the patient’s fear of pain and psychological burden, adherence to rehabilitation training is often compromised. In this paper, the IMB model rehabilitation exercise measures were used to intervene in patients treated with total knee arthroplasty, in order to explore its effect on improving the compliance of rehabilitation exercises and knee joint function.

The results of this study showed that the functional exercise compliance scores of the observation group were significantly higher than those of the control group at 1 and 3 months after surgery. Correspondingly, the Knee HSS scores, KOOS and range of motion of knee joint in the observation group showed greater improvement over time, indicating enhanced functional recovery. Additionally, the observation group demonstrated significant advantages in pain scores and 6-min walk distance (6MWD). All these improvements reflect increased walking independence, better ability to perform daily activities, and reduced risk of postoperative complications such as joint stiffness and muscle atrophy. Similar to the results of this study, the IMB model has been shown to help patients understand their needs and social support in postoperative knee arthroplasty rehabilitation, thereby improving treatment motivation and adherence (17). Studies have shown that the IMB model has achieved good intervention results in clinical nursing, especially in continuous nursing. It has solved the problem of incomplete information acquisition through methods such as telephone follow-up, and has significantly improved patients’ rehabilitation self-efficacy scores, functional exercise compliance scores, and knee joint function (14). In motivational interviews, the rehabilitation program can be designed to better meet the actual needs of patients by gaining a deeper understanding of their rehabilitation expectations, personal values, and the importance of rehabilitation exercises to their lives (18). Moreover, the model emphasizes the development of behavioral skills, such as goal-setting, self-monitoring, time management, and proper exercise techniques. These skills empower patients to overcome barriers to rehabilitation (e.g., pain, fatigue, fear of movement), leading to more regular and effective engagement with functional exercises. The IMB-based rehabilitation program thus offers multidimensional support—delivering relevant health information, stimulating motivation, and enhancing self-regulatory and physical skills. By accounting for individual differences and tailoring interventions to patients’ psychological and behavioral needs, this approach can substantially improve exercise adherence and promote better joint outcomes, including increased range of motion, reduced pain, and greater functional independence (19).

Because patients treated with knee arthroplasty have a fear of movement after surgery, which is caused by fear of knee dislocation, pain, and other factors, and some middle-aged and elderly patients do not accept information about active exercise, they cannot fully grasp the key points of functional exercise during the hospital stay, which will have a certain impact on knee function while reducing compliance with functional exercise (20). Compared with the control group, the patients in the observation group had significantly better improvement in knee function at 1 and 3 months after surgery according to the knee joint function scores. This finding aligns with that of Zhang and Li (21), who reported significantly higher knee function scores at 4 and 6 weeks postoperatively in patients managed with an IMB-based nursing program. Similarly, Chen et al. (17) found that IMB-guided continuous care improved self-care compliance and quality of life in postoperative knee arthroplasty patients. While these previous studies primarily focused on short-term improvements or subjective indicators, the current study extends the evidence base by incorporating both compliance and objective knee function assessments across multiple time points, offering a more comprehensive understanding of the IMB model’s sustained benefits. Moreover, by emphasizing individualized rehabilitation design—taking into account the severity of joint disease, patient background, and psychological readiness—this study highlights the versatility and scalability of the IMB model in real-world clinical settings. Through adequate information transfer, patients are able to understand the principles and effects of knee rehabilitation exercises, and thus are more motivated to participate in the rehabilitation program (22). The IMB model focuses on motivation, by understanding the patients’ expectations, values, and personal motivations for rehabilitation exercises, the rehabilitation exercise program can better meet the actual needs of the patients (23). Based on the individualized principle of the IMB model, the rehabilitation exercise program should be designed according to the individual differences of the patient, taking into account the degree of knee disease, life background, and psychological condition of the patient. A comprehensive rehabilitation program provides patients with comprehensive support and is expected to improve their quality of life and better adapt to the rehabilitation process while improving knee function (24).

Although rehabilitation protocols may vary across different centers, the IMB model provides a flexible and structured framework that can be adapted to diverse clinical settings. Rather than altering the core rehabilitation procedures, the IMB model enhances patient participation by targeting psychological and behavioral determinants of recovery. In our study, this model was implemented using existing healthcare staff and standardized educational materials, without requiring additional personnel or significant financial investment. This suggests that the IMB model is a cost-effective and scalable approach that can be integrated into routine care to improve rehabilitation adherence and outcomes.

Unlike previous studies that predominantly applied the IMB model in post-discharge or community-based rehabilitation settings, this study represents a novel contribution by initiating the IMB-based intervention during the inpatient period following TKA. This early engagement allowed patients to receive tailored information, motivation enhancement, and behavioral skill training at a critical window of recovery, potentially improving their psychological readiness and functional participation from the outset. Furthermore, the study employed a longitudinal design assessing both compliance and knee joint function at multiple postoperative time points, offering a more comprehensive and dynamic understanding of the intervention’s impact. Additionally, by integrating the principles of individualized care—accounting for patient-specific clinical, psychosocial, and motivational factors—this study demonstrates the feasibility and effectiveness of a personalized IMB-based rehabilitation program within routine orthopedic nursing practice. These methodological innovations not only extend the applicability of the IMB model but also suggest that early, structured, and patient-centered interventions may yield superior rehabilitation outcomes in TKA populations.

This study has several limitations. First, due to time and resource constraints, the sample size was relatively small and participants were recruited from a single center, which may limit the generalizability of the findings. Second, the follow-up period was limited to 3 months postoperatively, and long-term effects of the IMB-based intervention on functional outcomes and exercise adherence remain unclear. Third, as participants and outcome assessors were not blinded, there is a potential risk of performance and assessment bias. Moreover, key outcomes such as functional exercise compliance and knee function were measured using self-reported scales or clinician-rated instruments, which may be subject to recall bias or subjective interpretation. Finally, although the IMB model emphasizes the integration of information, motivation, and behavioral skills, its application in diverse cultural and social settings remains complex. Factors such as patients’ health literacy, family support, and access to rehabilitation resources may influence the effectiveness of the intervention, and further research is needed to explore culturally tailored strategies for enhancing rehabilitation engagement.

This study demonstrated that a rehabilitation training program based on the IMB model significantly improved postoperative functional exercise compliance and knee joint function in patients undergoing TKA, compared to conventional nursing interventions. These findings suggest that integrating the IMB model into perioperative care may enhance patient engagement and optimize rehabilitation outcomes. The model’s emphasis on personalized information delivery, motivational support, and behavioral skill development appears particularly effective in addressing common barriers to adherence. However, further multi-center studies with larger sample sizes and longer follow-up periods are warranted to confirm the long-term benefits and scalability of this approach. Future research should also explore culturally sensitive adaptations of the IMB model to ensure its effectiveness across diverse patient populations.

## 5 Conclusion

In summary, the rehabilitation exercise intervention based on the IMB model significantly improved functional exercise compliance and knee joint function in patients undergoing total knee arthroplasty. Compared to the control group, the observation group showed notably higher compliance scores and Knee HSS scores at 1 and 3 months postoperatively, indicating a moderate to substantial improvement in functional outcomes. However, the study’s conclusions should be interpreted with caution due to certain limitations, including a relatively small sample size, short follow-up period, reliance on subjective assessment scales, and the single-center design, which may limit the generalizability of the findings. Future large-scale, multi-center, and long-term studies are recommended to further validate the effectiveness and applicability of the IMB-based rehabilitation approach in diverse clinical settings.

## Data Availability

The original contributions presented in this study are included in this article/Supplementary material, further inquiries can be directed to the corresponding authors.
