# Supplementary material for: Effects of IMB model-based rehabilitation on exercise compliance and knee function after total knee arthroplasty: a randomized controlled trial
Source: Front Med (Lausanne). 2025 Dec 15;12:1619435. doi: 10.3389/fmed.2025.1619435 (PMC12745423; doi:10.3389/fmed.2025.1619435)
Supplement: Supplementary file 1 [file Table_1.docx]

**Table S1.** Differences of Rehabilitation Programs between the Two Groups

| Content | Observation group | Control group |
| --- | --- | --- |
| Rehabilitation plan | Personalized stage training based on the IMB model | No active training plan |
| Intervention team | Multidisciplinary collaboration team  (1 primary nurse, 1 psychologist, 1 rehabilitation therapist, 1 orthopedic surgeon, and 1 orthopedic specialist nurse) | Primary nurse |
| Information support | Personalized exercise plan; multimedia resources; Establish a community support system; | Standard health education and passive learning for family members |
| Motivational interview | Building a relationship of trust; Open-ended questions and listening; Emphasizing personal values | None |
| Follow-up management | Regular follow-up and Remote Communication (such as telephone, email, or online chat) | Only education at discharge |
| Effect indicators | Physiological indicators+psychological indicators+psychological indicators | Basic physiological indicators |
